# Systematic screening for cervical cancer in Dakar region: prevalence and correlation with biological and socio-demographic parameters

**DOI:** 10.1186/s13027-020-00290-y

**Published:** 2020-04-22

**Authors:** Dominique Diouf, Gora Diop, Cheikh Ahmadou Tidian Diarra, Aminata Issa Ngom, Khadija Niane, Moussa Ndiaye, Sidy Ka, Oumar Faye, Ahmadou Dem

**Affiliations:** 1 Laboratory of cytogenetic and reproductive biology, Hopital Aristide-Le-Dantec, Pasteur Avenue, PO Box 3001, Dakar, Senegal; 2 Institut-Juliot-Curie, Hospital Aristide Le Dantec, Pasteur Avenue, PO Box 3001, Dakar, Senegal; 3 Laboratory of anatomy and pathology, Principal military Hospital of Dakar, Nelson Mandela Avenue, PO Box 3006, Dakar, Senegal; 4 Cancer Studies and Research Group in Senegal (GERCS), Cheikh Anta Diop Avenue, PO 5005, Dakar, Senegal; 5 Department of Animal Biology, Faculty of Science and Technology, Postulant Unit of Genetic, Genomic and Bioinformatic of Infectious Diseases, Cheikh Anta DIOP University, PO Box 55, Cheikh Anta Diop Avenue, Dakar, Senegal; 6 Immunology & Biology of infectious diseases Unit, Institut Pasteur Dakar, 36, avenue Pasteur, PO Box 220, Dakar, Senegal

**Keywords:** Cervical cancer, Dysplasia, Epidemiology, Prevalence, Senegal

## Abstract

**Background:**

Cervical cancer is a major public health problem. In 2018, globally 569,847 cervical cancer were diagnosed and 311,000 deaths were projected due to this preventable disease. Worldwide, therefore, the cervical cancer disease ranks as the fourth most frequently diagnosed cancer and the fourth leading cause of cancer death in women in 2018.

The high rate of dysplasia in Senegal and the absence of well-organized screening programs informed this study, which aims to determine the prevalence of cervical dysplasia and its relationship to biological and socio-demographic characteristics.

**Methods:**

This study is based on 1000 conventional smears collected during routine cervical cancer screening at the Gaspard Camara Health Center and the Histology - Embryology and Cytogenetics Laboratory of the Cheikh Anta DIOP University in Dakar. The smears were read according to the Bethesda and Richart systems. However, all data were returned to the Bethesda system using the correspondence table between the different classifications of squamous cell lesions of the cervix. Some of the patients with abnormal smears had colposcopy and if necessary a biopsy. Other patients with low-grade lesions were recommended to have their smears resumed in 6 months or 1 year later.

**Results:**

Cytological analysis was performed for 1000 patients aged 16 to 82 years (mean age = 41 ± 11.16). Among these, 176 patients had abnormal smears, 23 had Atypical Squamous Cells of Undetermined Significance (ASCUS), 143 had a low-grade lesion, 9 had a high-grade lesion and 1 had carcinoma. Among the remaining 822 patients, cytological analysis revealed no suspected malignant lesions, but 623 among them had dystrophy and 2 were unsatisfactory. Among patients with abnormal smears, 104 patients (23 ASCUS + 71 low grade + 9 high grade + 1 carcinoma) had performed colposcopy, 40 of whom had normal colposcopy and 64 had abnormalities. Sixty-four (64) biopsies were performed. Four (4) were not satisfactory. However, for 26/60 biopsies, the histology was normal, 21/60 had a low grade, 11 displayed a high grade and only 2 had carcinoma. Among the 176 patients with abnormal smears, 72 low-grade patients had undergone cytological examination 6 months to 1 year later to determine the persistence, regression or progression of low-grade dysplasia. During follow-up, persistence was observed in 25% (*n* = 18) of cases, progression to High-grade squamous intraepithelial lesion (HSIL) was detected in 2.78% (*n* = 2), while 72.22% (*n* = 52) of the patients experienced regression.

**Conclusion:**

In this study, the prevalence of abnormal smear was 17.60% for cytology. Meanwhile, the Colposcopy and histology confirmed just 3.40%. These results underline the interest and need for a review of the discrepancies observed between pathologists.

## Introduction

According to the latest estimates, cervical cancer remains the fourth most frequently diagnosed cancer and the fourth leading cause of cancer death in women in 2018, with an estimate of 570,000 cases and 311,000 deaths [1].

Most cases of cervical cancer occur in developing countries where control and screening programs remain ineffective. In contrast, in developed countries, implementation of programs has led to a significant reduction in incidence and mortality [2–4]. Since the introduction of organized cervical screening in the 1960’s in the U.S., incidence and mortality from cervical cancer have declined by 75%, however, this decline is not uniform [5].

In Africa, cervical cancer is the most common cancer among women, with a high prevalence in the 15 to 44 age group. Most of them belong to the most disadvantaged population [6, 7]. More than 90% of all observed cervical cancer cases are related to persistent high-risk human papillomavirus (HPV-HR) infection, resulting in precancerous lesions that can progress to invasive cervical cancer if left untreated [6, 8].

About 40 genotypes infect the genital tract and are classified according to their oncogenic potentials. The high-risk types are HPV-16 and HPV-18. They are responsible for about 70% of cervical cancer cases [9, 10].

Cervical cancer is on the increase in sub-Saharan Africa, with more than 75,000 new cases and 50,000 deaths per year which is aggravated by HIV infection [11].

In Senegal, cervical cancer is the leading cause of female cancer deaths with an estimated 1876 cases diagnosed annually, of which 1367 cases (72.90%) are fatal [12].

The objective of this study, which is strictly limited to Dakar region, was to provide a prevalence of cervical dysplasia in Senegal from 1000 systematically screened patients and to correlate the results with biological and socio-demographic characteristics. Further, we aimed to examine the concordance between cytological and histological analysis and to evaluate persistency, progression and regression in patients with low-grade dysplasia.

## Material and methods

### Study population and sample collection

From January 2015 to December 2018, 1000 cervical smears were collected and analyzed from Senegalese women aged 16 to 82 years, who participated in a voluntary screening at the Histology - Embryology and Cytogenetics Laboratory of the Cheikh Anta Diop University of Dakar (UCAD) (*n* = 357) and the Gaspard Camara Health Center (*n* = 643) (Table 1). Informed consent was obtained from all patients. The study subjects were interviewed using a structured questionnaire that took into account socio-demographic and reproductive characteristics such as: age, education level, occupation, marital status, place of residence, contraceptive use, depigmentation, pregnancy and parity (Table 1). The local research ethics committee of Cheikh Anta DIOP University (UCAD) approved this study under the Protocol 0194 l 2016 / CER/UCAD.

**Table 1:** Socio-demographic and reproductive characteristic of participants (*n* = 1000)

Socio-demographic characteristics	Values N (%)
**Age (yrs)**	
16-**29**	162 (16.20)
30-**39**	299 (29.90)
40-**49**	321 (32.10)
**50–59**	161 (16.10)
**Above 60**	57 (5.70)
**Mean age ± SD**	41 ± 11.16
**Education**	
Low level	876 (87.60)
High level	124 (12.40)
**Education level**	
No formal education	289 (28.90)
Primary level	273 (27.30)
Secondary level	314 (31.40)
Higher level and above	124 (12.40)
**Parity**	
0	99 (9.90)
1-2	327 (32.70)
>2	574 57.40)
**Gestity**	
0	67 (6.70)
1-2	265 (26.50)
>2	668 (66.80)
**Contraceptive use**	
No	403 (40.30)
Hormonal	491 (49.10)
Non hormonal	106 (10.60)
**Skin lightning**	
No	426 (42.60)
Yes	574 (57.40)
**Occupation**	
Unemployed	549 (54.90)
Employed	150 (15.0)
Self-employed	301 (30.10)
**Marital status**	
Single	73 (7.30)
Divorced/Separated	38 (3.80)
Widow	33 (3.30)
Married (husband monogamous)	523 (52.30)
Married (husband polygamous)	333 (33.30)

Data shown are number (%) except otherwise specified, SD: standard deviation

### Cytology

In Senegal, the conventional method remains the Papanicolaou test, which is the most widely used method in screening programs [13]. However, the access to the Pap test is very limited except in certain highly urban settings.

All women were screened for abnormal cervical cytology using the conventional Papanicolaou smear. The cells were collected with a spatula, placed on a glass microscope slide and then, fixed with a spray. The smear readings were taken using the Bethesda and Richart systems. The concordance between the different classifications of squamous cell lesions of the cervix made it possible to bring all the results of the reading back to the Bethesda system, represented as follows: Normal, Atypical Squamous cells of undetermined significance (ASCUS), Low-grade squamous intraepithelial lesion (LSIL), High-grade squamous intraepithelial lesion (HSIL) and carcinoma [14].

### Histology, colposcopy and control smears

Following the cervical smear, some patients with low-grade dysplasia were advised to take a control smear 6 months or a year later, while others were directly referred for colposcopy. Patients with abnormal colposcopy had undergone a biopsy. Those whose control smears revealed persistent dysplasia underwent colposcopy and biopsy if necessary at the cancer department of Aristide le Dantec Hospital (Curie-HALD). For those whose smears revealed HSIL, colposcopy and, if necessary, a biopsy was performed. The samples were analyzed at the histopathology laboratory of “Hôpital Principal” or at Aristide le Dantec Hospital to confirm or invalidate the grade of the lesion revealed by the smear. Patients with LSIL who had not undergone colposcopy were received at the Gaspard Camara Health Center for a control smear.

### Statistical analysis

Statistical assessment was carried out using *Statistical Package for the Social Sciences (SPSS*) version 20.0 (IBM Corp., Armonk, NY, USA). Prevalence was determined using percentages and cross-tabulations were used to describe the association of potential risk factors with an abnormal cytological result. The χ2 test was used for the qualitative variables. Concordance between cytology and histology was tested using Kappa statistics.

The relationship of abnormal cervical cytology with potential risk factors: Age, contraceptive use, education, parity, gestity, skin lightening was analyzed using a multivariate logistic regression. The results were considered statistically significant if the *p*-value of logistic regression coefficient were < 0.05.

## Results

### Socio-demographic characteristics

Table 1 summarizes the socio-demographic characteristics of 1000 patients included in the study. In summary, the range of patient age is from 16 to 82 years, with an average age of 41 ± 11.16 years. The age group with the largest number of participants was the 40–49 age bracket with 32.10% enrolled patients. The percentage of those with no form of education was 28.90 and among the literates, 27.30% could barely read and could not write.

Among the participants, 66.80% had 2 or more pregnancies and 57.40% had 2 or more children. Regarding contraception, 49.10% used the hormonal method, 10.60% used non-hormonal contraception, while 40.30% did not use contraception.

### Cytology

The smear analysis was performed on 1000 samples, and two were unsatisfactory. Among the total smears, 176 had abnormal pap smear, 822 were normal or dystrophic. Smear abnormalities included 23 ASCUS, 143 low-grade squamous intraepithelial lesions (LSIL), 9 high-grade squamous intraepithelial lesions (HSIL) and 1 squamous cell carcinoma (Table 2). The highest rate of abnormal smears was noted in older women (50–59 years old; 24.80%). The mean age of women with a normal smear was 41 ± 11.03 years, while the mean age of women with an abnormal smear was 43.08 ± 11.55 years.

**Table 2:** Cytology reports

Tests (Cytology)	Values N(%)
Unsatisfactory smears	2
Normal smears	199 (19.93)
Inflammatory smears	623 (62.30)
ASCUS	23 (2.30)
Low grade (LSIL)	143 (14.32)
High grade (HSIL)	9 (0.90)
Carcinoma	1 (0.10)
Total cytology	1000 (100)

The data displayed is in number (%). HSIL: high-grade squamous intraepithelial lesion; LSIL: low-grade squamous intraepithelial lesion; *ASCUS* Atypia of squamous cells of undetermined significance

### Histology and colposcopy

Among the patients with low-grade lesions (LSIL) and patients with ASCUS, 104 had performed colposcopy among which, 40 were normal and 64 had abnormalities. Further, biopsies were carried out on those displaying abnormalities. Four (4) were unsatisfactory, 12 had normal histology, 10 patients had cervicitis, 4 had a polyp, 21 had a low-grade lesion, 11 had a high-grade lesion and 2 had carcinoma (Table 3).

**Table 3:** Histology reports

Tests (Histology)	Values N(%)
Unsatisfactory samples	4
Cervicitis	10 (16.66)
Polyps	4 (6.66)
Normal	12 (20)
Low grade (LSIL)	21 (35)
High grade (HSIL)	11 (18.33)
Carcinoma	2 (3.33)
Total biopsy	64 (100)

The data displayed is in number (%). *HSIL* High-grade squamous intraepithelial lesion; *LSIL* Low

grade squamous intraepithelial lesion

### Multivariate correlation of different variables with cytology results

Table 4 shows the distribution of dysplasia according to the different parameters studied. The percentage of abnormal smears was higher among multiparous women (13.42%), those who used contraception (12.02%) and those who practiced depigmentation (9.01%) in that order. Statistical analysis (logistic regression) of the data showed that age and contraceptive use had significant values with a *p*-value of *p* = 0.0024 and *p* = 0.005 respectively.

**Table 4:** Multivariate analysis (logistic regression) of different variables with cytology report (*N* = 998)

Characteristics	Cervical Cytology		*P*-value
	Abnormal (*n* = 176)	Normal (*n* = 882)	
**Age (yr)**			0.0024
< 45	50 (5.01)	288 (28.85)	
≥ 45	126(12.62)	534 (53.50)	
**Education**			0.82
High Level	21(2.10)	103(10.32)	
Low Level	155(15.53)	719(72.04)	
**Parity**			0.99
0–2	42(4.20)	209(20.94)	
> 2	134 (13.42)	613 (61.42)	
**Gestity**			0.53
0–2	42(4.20)	207(20.74)	
≥ 2	134(13.42)	615(61.62)	
**Skin lightning**			0.05
Yes	90(9.01)	482(48.29)	
No	86(8.61)	340(34.06)	
**Contraceptive use**			0.005
Users	120(12.02)	473(47.39)	
Non-users	56(5.61)	349(34.96)	
**Marital status**			0.30
Married	155(15.53)	699(70.04)	
No Married	21(2.10)	123(12.32)	

Data shown are number (%) not otherwise specified

### Persistency, progression and regression of LSIL

In this study, 72 patients with low-grade dysplasia were referred to the Gaspard Camara Health Center for a control smear 6 months or 1 year later to determine the persistence, regression or progression of dysplasia. During follow-up, persistency was observed in 25% (*n* = 18) of cases, progression to HSIL in 2.77% (*n* = 2) and regression in 72.22% (*n* = 52) of patients (Table 5).

**Table 5:** Control smear results for LSIL patients

Cytology		Persistency		Progression		Regression	
*n*	***%***	*n*	***%***	*n*	***%***	*n*	***%***
**72**	100	18	25	2	2.78	52	72.22

*LSIL* Low-grade squamous intraepithelial lesions

## Discussion

With an estimated 570,000 cases and 311,000 deaths in 2018 worldwide, this disease ranks as the fourth most frequently diagnosed cancer and the fourth leading cause of cancer death in women. The highest regional incidence and mortality rates are seen in Africa with rates elevated in Southern Africa (43.1 per 100.000), Eastern Africa (40.1 per 100.000), and Western Africa (29.6 per 100.000) [1].

Senegal ranks 17th in the world for the incidence of cervical cancer, which is the leading cause of cancer death among women in the country [1, 15].

The estimated participation rate for cervical cancer screening in Senegal is very low (6.90% of women aged 18 to 69), especially in rural areas and among older groups (only 1.90% of women over 40). There is no reliable estimate of the prevalence of cervical dysplasia or risk factors for cervical dysplasia specific to Senegal in rural areas [15, 16].

In our study, the average age of the patients was 41 ± 11.16 years and the most represented age group in our cohort was 40-49 years (32.10%). It should be noted that 66.12% of the patients were between 36 and 82 years of age. These data are similar to those of previous studies such as those of Sy-Diallo et al. [17] and Mbaye et al. et al. [18], which found an average age of 41.1 ± 11 years and 42.1 years respectively. However, this average age is higher than what was obtained by DEM et al. [19] in a study of squamous cell carcinomas conducted at Aristide le Dantec Hospital.

The majority of the patients had no formal education or are of low academic level (87.60%) and more than half of the participants were multiparous (57.40%) with at least 2 children. Nearly 60% of patients were using birth controls and 57.40% were depigmenting (Table 1). These socio-demographic characteristics of our cohort differ from those of the study conducted by Somé et al. [20] where patients were younger with 69% being under 45 years of age. In addition, they did not perform depigmentation, however, most patients were multiparous.

The prevalence of dysplasia estimated by cytology was 17.60%, which is similar to the results of previous studies such as those conducted by Afoutou et al. (16.34%), Diallo et al. (21.03%), Xi LF et al. (20.10%) [21–23]. However, the rates found by Somé et al. (5.20%) and SY-DIALLO et al. (7.50%) were lower [17, 20]. These differences could be explained by the origin of the patients who, in our study, were concentrated in the urban areas of Dakar, unlike the studies conducted by Somé et al. [20] and Sy-Diallo et al. [17] who recruited their patients from rural areas.

This suggests that the rate of dysplasia is higher in urban than in rural areas. Another cofactor to be considered is the age of the patients. In the study of Somé et al. [20], patients were younger (69%) and are under 45 years of age). However, most studies conducted in Senegal confirm a relationship between an increase in the prevalence of dysplasia and the age of the patients [17, 19, 21].

In this study, age (*p* = 0.0024) and contraceptive use (*p* = 0.005) parameters were identified as being associated with the occurrence of dysplasia or carcinoma. Other parameters such as depigmentation (*p* = 0.05) were associated but not statistically significant. We did not found a link between parity, education, as well as marital status, and the occurrence of dysplasia. These results are consistent with results of studies conducted by Kassa RT. [24]; Zidi et al. [25]; Iverson et al. [26]; Roura et al. [27]; Vaisy et al. [28] that found a relationship between age, contraceptive use and the occurrence of dysplasia or cancer. However, these results contrast with those found by Paweena et al. [29]; Somé et al. [20] who did not identify contraception and age as a risk factors. On the other hand, Niresh et al. [30] found an association between age and cervical cancer but not with contraception.

Among the 176 patients with dysplasia or cancer, 104 underwent colposcopy and 72 had a control smear. Among the 104 patients, 40 had normal colposcopy while the remaining 64 had a biopsy (4 biopsies were unsatisfactory). Biopsy analyses showed 34 histological abnormalities out of 60 (low-grade intraepithelial lesions = 21, high-grade intraepithelial lesions = 11, Carcinoma = 2).

The poor Kappa coefficient (k ≤ 0.00) found in our study shows a lack of cyto-histological agreement among LSILs. These findings are lower than that found by Manzo-Banales et al. [31]; Melinte-Popescu et al. [32]; Oh et al. [33]; Islam et al. [34]; Önder et al. [35]. However the kappa coefficient found in this current study is similar with that found in the study conducted by Dasari et al. [36].

These differences could be explained in part by the duration patients with abnormal cytology delayed before being called for a biopsy, sometimes with 6 months to 1 year or more, which could lead to a regression of LSIL largely or a progression to HSIL. This difference could also be explained by technical problems related to the quality of the sample or errors related to reading or staining. These shortcomings could also be justified, in part, by the lack of funding to cover the costs of histology and the availability of colposcope.

Previous studies have shown that the use of simple colposcopy at follow-up has low sensitivity [37]. It has been shown that 70% of LSIL lesions regress by themselves; however, 10% progress to HSIL [38, 39].[. Similarly, Shaki Ö et al. [40] found somewhat similar results with persistence of 22%, progression of 9% and regression of 68%. These results are in agreement with those found in our study where LSIL cases decreased by 72.20% and the progression rate are 2.70%. Regarding the persistence of dysplasia, which is one of the main causes of cancer occurrence, it is 25% in our study. However, other authors have found different results. Indeed, Cortes et al. [41] observed a spontaneous regression of LSIL in 50% of patients and a progression of HSIL in 6% during the 2 years of follow-up [42], and found an 18% progression, 74% persistence and 8% regression in cases of low-grade lesions (LSIL). While Pretorius et al. [43] found a 57% regression in LSIL cases, 32% persistence and 11% progression in HSIL. These differences in the results obtained could be justified by the duration of the follow-ups, which may vary from one author to another.

Following all these observations, it is certain that Papanicolaou smear repetition is recommended for LSIL patients [44, 45]. Those with persistent LSIL undergo cryotherapy; high-grade profiles undergo conization or hysterectomy, while those with carcinoma have been treated with radiotherapy and/or chemotherapy.

This study has some limitations: The study was limited mostly to patients residing in the urban area of Dakar, which does not provide information at the national level. Other limitations are the long time between cytology, histology experiments and the heterogeneous reading of pap smears. According to cyto-pathologists, the reading is done according to Richart or Bethesda method. To correct this, all the data obtained were returned to the Bethesda system, using the agreement table between the different classifications of squamous cell lesions of the cervix [14].

## Conclusion

In conclusion, the observation of this study raises a concern about the burden of cervical cancer in Dakar, where reliable statistics on cervical cancer are limited, despite this study showing the high prevalence of cervical dysplasia’.

Based on cytological analysis, we found an overall prevalence of 17.60% of abnormal smears. However, histology and colposcopy confirmed 3.40% of abnormality on all smears, which shows a poor concordance between cytology and histology that could be linked to the high rate of regression of LSIL before the biopsy.

The study of cofactors in the occurrence of cervical dysplasia or cervical cancer showed that patients who used contraception were more at risk of developing cervical dysplasia. The study shows also that older women have the highest risk of developing cervical cancer, and most low-grade lesions (LSIL) spontaneously regress while a small proportion progresses to high-grade dysplasia. The authors believe that new methods for evaluating cervical pathologies, such as liquid cytology, will reduce false negatives and positives and eliminate current uncertainties. In addition, the introduction of biomarkers associated with cytology such as P16ink4A/Ki67 would reduce the number of patients referred for histology while providing better diagnostic accuracy.

## Data Availability

The datasets used and/or analyzed during the current study are available from the corresponding author upon reasonable request.

## Abbreviations

ASCUS: Atypical squamous cells of undetermined significance
HSIL: High-grade squamous intraepithelial lesion
LSIL: Low-grade squamous intraepithelial lesion
HPV-HR: High-risk human papillomavirus (HPV-HR)
HIV: Human immuno-deficience virus
UCAD: University cheek anta diop
CER: Centre d’études et de Recherche
HALD: Hopital aristide le dantec
SPSS: Statistical package for the social sciences
RR: Relative risk
CI: Confidence interval
